# Transdermal Delivery of Adriamycin to Transplanted Ehrlich Ascites Tumor in Mice

**DOI:** 10.3390/pharmaceutics5030385

**Published:** 2013-07-10

**Authors:** Masataka Shiozuka, Yoshiaki Nonomura, Ryoichi Matsuda

**Affiliations:** Department of Life Sciences, Graduate School of Arts and Sciences, The University of Tokyo, 3-8-1 Komaba, Meguro-ku, Tokyo 153-8902, Japan; E-Mails: cmuscle@mail.ecc.u-tokyo.ac.jp (M.S.); cnono@mail.ecc.u-tokyo.ac.jp (Y.N.)

**Keywords:** (trans)dermal drug delivery, thioglycolate, adriamycin

## Abstract

There is considerable interest in the skin as a site of anti-cancer drug application. Nevertheless, the skin poses a formidable barrier to drug penetration, thereby limiting topical and transdermal bioavailability. However, we previously showed that a thioglycolate-based depilatory agent increases the drug permeability of mouse skin. In the present report, we investigated the skin permeability and efficacy of the anti-cancer drug adriamycin increased when administered transdermally to mice in combination with a thioglycolate-based depilatory agent. Adriamycin in combination with depilatory treatment reduced Ehrlich tumor growth in hairless mice about the weight and size of harvested tumors. In addition, our delivery method for adriamycin increased the therapeutic effectiveness of this agent by decreasing toxicity. Moreover, measurement of adriamycin autofluorescence revealed that topically applied adriamycin penetrate the dermis after depilatory agent treatment. These results indicate that the transdermal delivery of anti-cancer drugs is feasible by handy pretreatment of the skin with a thioglycolate-based depilatory agent.

## 1. Introduction

Oral and intravenous administration are the two main drug delivery routes for anti-cancer drugs. However, the skin is the largest organ in the body and an obvious route for both local and systemic drug delivery. Transdermal drug delivery possesses several advantages over oral and intravenous drug administration [1], in that it: (1) bypasses gastrointestinal incompatibility and the hepatic “first-pass” effect; (2) reduces side-effects through optimization of blood concentration-time profiles; (3) involves patient-activated/patient-modulated delivery, which enhances patient compliance; (4) enhances target specificity; and (5) reduces medical treatment costs. Transdermal drug delivery systems offer many advantages over conventional administration routes [2]. Given the low permeability of external molecules through the skin, it remains a minor portal of entry for drugs in the clinical setting [3]. Thus, the (trans)dermal delivery of anti-cancer drugs may be useful in the clinical settings if the skin permeability of drugs can be increased.

We previously showed that the skin permeability of gentamicin increased when combined with a thioglycolate-based depilatory agent [4]. Ultrastructural studies revealed that alteration and expansion of intracellular spaces in the epidermis and dermis were responsible for the increase of drug permeability of depilatory agent-treated skin.

Adriamycin (doxorubicin hydrochloride) is an anthracycline antibiotic that is commonly used in the treatment of a wide range of cancers, carcinomas and soft tissue sarcomas. Various approaches aimed at decreasing the resistance of skin to drug penetration have been investigated [5]. For example, Herai *et al.* [6] found that the penetration enhancer monoolein significantly increased the *in vitro* skin permeation and retention of adriamycin in the stratum corneum. Besides, Ta *et al.* [7] reported that the incorporation of adriamycin in chitosan/dipotassium orthophosphate hydrogel not only significantly inhibited the growth of osteosarcoma, osteolysis and lung metastasis, but also reduced the side-effects of adriamycin in mice. Moreover, Han *et al.* [8] reported that although transdermal adriamycin delivery is enhanced by liposomal formulations, topical applications have a few limitations with regard to delivery capacity and speed. The liposome-mediated delivery of adriamycin proceeds through follicular routes and has a significant synergistic effect in combination with iontophoresis. Further, Tamer *et al.* [9] reported that the active targeting of the long-circulating liposomal preparations of adriamycin with the anti-tumor antibody presents an attractive opportunity to further control the *in vivo* behavior of the original drug and improve its tolerability. Thus, vesicular systems such as liposomes, niosomes, ethosomes and elastic, deformable vesicles provide an alternative for improved skin drug delivery [10].

Here, we investigated whether thioglycolate-based depilatory agent-treatment increases the skin permeability of adriamycin and its anti-tumor activity for Ehrlich carcinoma cells grown underneath the skin. The anti-cancer effects of adriamycin with the depilatory treatment were evaluated by the inhibition of tumor growth, which was determined by comparing the weight and size of harvested tumors from hairless mice after 21-day treatment. Besides, we compared the anti-cancer effects of adriamycin of two different doses and intervals during 14-day treatment. Moreover, to determine the efficacy of our depilatory method, the distribution of adriamycin applied as a cream to hairless mice were examined based on adriamycin autofluorescence by fluorescence microscope.

## 2. Experimental Section

Adriamycin cream was prepared by mixing Adriacin (Kyowa Hakko Kirin Co., Ltd., Tokyo, Japan) with White Ointment (Nikko Pharmaceutical Co., Ltd., Gifu, Japan) using a planetary centrifugal mixer, AR-100 (Thinky INC., Tokyo, Japan). The final concentration of adriamycin was 0.2 or 0.6 mg/g of cream. Five-week-old female hairless mice (HR1; body weight, approximately 20 g) were obtained from Japan SLC, Inc. (Shizuoka, Japan) and were housed individually under controlled temperature and humidity conditions, and had free access to water and food. The present study was approved by Animal Ethics Committee of the University of Tokyo.

Ehrlich carcinoma cells were cultured in RPMI 1640 supplemented with 10% fetal bovine serum at 37 °C in a humidified 5% CO_2_ atmosphere. To prepare cells for transplantation into mice, exponentially growing cells were harvested, washed, and then resuspended in RPMI 1640. Ten million of Ehrlich carcinoma cells were transplanted subcutaneously into the backs of hairless mice. Three days after the transplantation, the mice bearing tumors that did not reached a diameter of 1 cm were excluded. The mice were randomly divided into 4 groups containing maximum 6 animals per group. 

Depilatory cream was obtained from Reckitt Benckiser Co., Ltd. (Tokyo, Japan) and was applied once every 3 days for 1 min to the mouse skin, which was then rinsed with warm water to remove the cream. Adriamycin cream (0.5 g) was then gently applied by rubbing onto the skin of the transplantation site daily for 21 days (a total of 7 treatments with depilatory cream were performed during this time). Treatment started 3 days after tumor cell transplantation when tumors reached a diameter of approximately 1 cm. At the end of the treatment, the mice were euthanized with an overdose of ether. Euthanasia and carcass disposal were performed in accordance with the institutional guidelines for animal care of the University of Tokyo. Solid tumors were washed with saline after being excised, and then weighed.

To examine the distribution of adriamycin, 10-µm cryosections of the tumor specimens were prepared and then examined under a fluorescence microscope (Axioplan, Carl Zeiss GmbH, Oberkochen, Germany) to visualize adriamycin, which has excitation and emission peaks of 488 nm and 556/582 nm, respectively. The obtained images were optimized for contrast and brightness using Photoshop CS5 software (Adobe Inc., San Jose, CA, USA).

## 3. Results and Discussion

**Figure 1:**
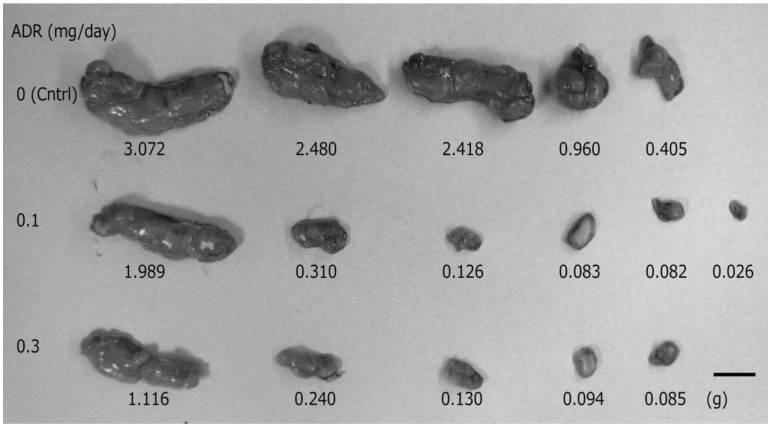
Effect of topically administered adriamycin on tumor growth in hairless mice. In mice treated with adriamycin cream (0.1 or 0.3 mg/day; *n =* 6, 5, respectively), the tumor size and weight (**middle** and **bottom** sections) were significantly smaller than those in the untreated control group (**upper** sections; *n =* 5). A higher dose of adriamycin (**bottom** section) slightly improved the inhibition of tumor growth. Scale bar = 1 cm.

Adriamycin is a potent anti-cancer agent that is clinically useful for the treatment of acute leukemias, malignant lymphomas, and carcinomas [11]. In the present study, we have shown that the use of depilatory treatment allows for the potential (trans)dermal delivery of this anti-cancer drug. We examined the anti-tumor effect of adriamycin administered topically to hairless mice bearing solid tumors that were induced by transplantation of Ehrlich’s carcinoma cells, which are widely used to form xenografts. Ehrlich carcinoma is a transplantable, poorly differentiated malignancy and grows in both solid and ascitic forms [12]. The anti-cancer effects of adriamycin with the depilatory treatment were evaluated by the inhibition of tumor growth, which was determined by comparing the weight and size of harvested tumors from untreated control and treated mice. As shown in Figure 1, transdermal treatment with adriamycin cream led to a reduction in tumor size compared to the untreated control group. After the transplantation, mice were randomly divided into 4 groups (0 mg/0.5 g TD, 0.1 mg/0.5 g TD, 0.3 mg/0.5 g TD, 0.1 mg/0.2 mL SC; *n =* 5, 6, 5, 6, respectively). Subcutaneous injection of the adriamycin solution into the transplantation site resulted in the death of all mice by the 15th consecutive day of treatment, whereas all mice administered adriamycin topically lived until the end of experiment. The effect of topically applied adriamycin cream (0.3 mg/0.5 g; *n =* 3) without depilatory treatment and adriamycin solution (0.1 mg/0.2 mL; *n=* 4) with depilatory treatment, were also examined by applying directly onto skin, but no reduction in tumor size was observed (data not shown).

**Figure 2:**
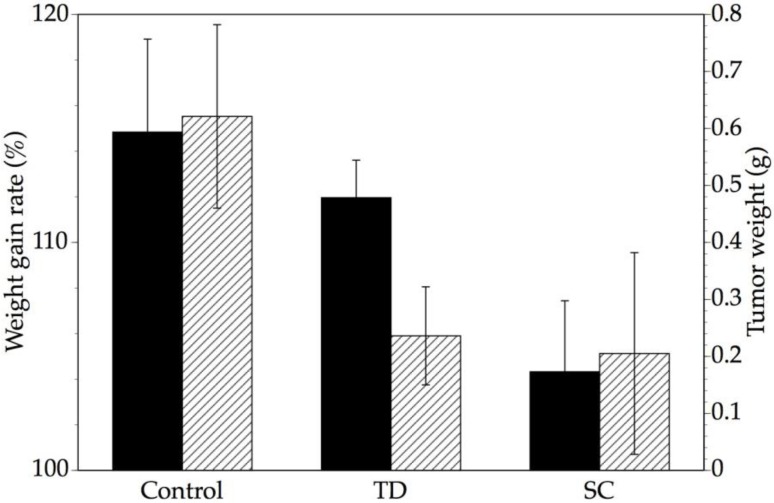
Comparison of transdermal and subcutaneous administration of adriamycin by weight gain rate and tumor weight. The body weight gain rate (black bars, left axis) and tumor weight (striped bars, right axis) of tumor-bearing mice transdermally administered (TD) and subcutaneously injected (SC) with adriamycin were compared (*n =* 4). The daily transdermal administration and the once every three days subcutaneous injection of adriamycin resulted in similar anti-cancer effects, as estimated by the tumor weight. However, transdermal adriamycin administration led to a higher weight gain rate in mice than subcutaneous injection (*p* < 0.05, Welch’s *t*-test), and was statistically similar to the level in the untreated control. Data are shown as the mean ± S.D..

We next compared the anti-tumor effects of adriamycin of two different doses and intervals during a 14-day treatment period. The daily topical administration (0.05 mg/0.5 g; *n =* 4) and intermittent (once every 3 days) subcutaneous injection of adriamycin (0.05 mg/0.2 mL; *n =* 4) had equivalent anti-cancer effects on tumor weight (Figure 2). In contrast, the transdermal administration of adriamycin resulted in statistically higher increase in the body weight gain rate (*p* < 0.05), which was used as a measure of toxicity, compared to subcutaneous injection, and was statistically similar to the control. These data suggest that the transdermal delivery of adriamycin can increase its therapeutic effectiveness by diminishing toxicity without affecting its anti-tumor activity. 

**Figure 3:**
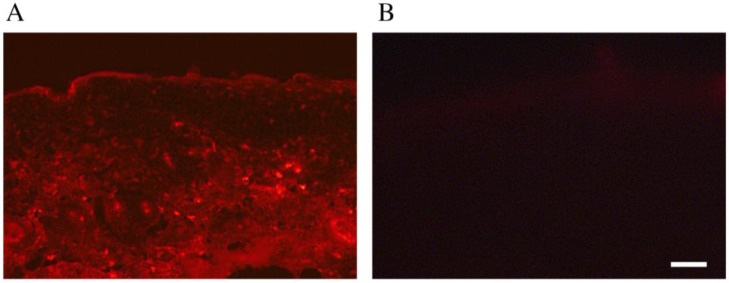
Distribution of transdermally administered adriamycin. Adriamycin cream was topically applied to the skin of hairless mice for three consecutive days with (**A**); or without (**B**) pretreatment with a depilatory agent. The distribution of adriamycin was then detected based on its autofluorescence. Adriamycin was observed in the dermis/fascia only after pretreatment with the depilatory agent. Scale bar = 10 µm.

We also examined the distribution of adriamycin following its application in cream form to the back area of hairless mice pre-treated with and without a depilatory agent. Adriamycin was detected in thin sections of the treatment area by its autofluorescence [13]. As shown in Figure 3, only topically administered adriamycin with the depilatory agent was observed in the epidermis to dermis and reached the underlying muscle layer.

We previously reported that liquid chromatography-tandem mass spectrometry (LC-MS/MS) can be used to validate the effect of a depilatory agent on the *in vivo* permeation of gentamicin [4]. LC-MS/MS analysis of the sera and muscle tissue extracts of hairless mice confirmed that the treatment drug was not detectable in the non-depilatory-treated group, but was present in the depilatory-treated-group. Using electron microscopy, we previously observed a large expansion of the intercellular gaps and extraordinary spaces in the basal and prickle-cell layers in depilatory agent-treated mice [4]. These results indicate that alteration and expansion of the intracellular spaces in the basal and prickle-cell layers of dermis may be due to the shrinkage of cells in those layers, which in turn leads to reduced resistance. Lee *et al.* [14] have shown that depilatory agents enhance transepidermal drug delivery by reducing the resistance of both the transcellular and intercellular routes of the stratum corneum. These findings, together with our present results for adriamycin, suggest that the combination of a skin impermeable drug with a depilatory agent increases drug penetration into the epidermis, not only for topical delivery but also for systemic delivery.

## 4. Conclusions

In conclusion, our results suggest that the pretreatment of skin with a depilatory agent increases the anti-tumor effect against Ehrlich solid tumor. Our present cancer treatment method involves the use of a thioglycolate-based depilatory agent to increase the permeability of the dermal surface and has proven to be more convenient and effective than injection. Thus, depilatory agent-treatment may be useful for the local application and systemic delivery of anti-cancer drugs. 
